# Optimal Stream Gauge Network Design Using Entropy Theory and Importance of Stream Gauge Stations

**DOI:** 10.3390/e21100991

**Published:** 2019-10-11

**Authors:** Hongjun Joo, Jiho Lee, Hwandon Jun, Kyungtak Kim, Seungjin Hong, Jungwook Kim, Hung Soo Kim

**Affiliations:** 1 Department of Civil Engineering, Inha University, Incheon 22212, Korea; 2 Department of Civil Engineering, Seoul National University of Science and Technology, Seoul 01811, Korea; 3 Department of Hydro Science and Engineering Research, Korea Institute of Civil Engineering and Building Technology, Ilsan 10223, Korea

**Keywords:** stream gauge network, entropy, station rating, Euclidean distance

## Abstract

Stream gauge stations are facilities for measuring stream water levels and flow rates, and their main purpose is to produce the data required to analyze hydrological phenomena. However, there are no specific criteria for selecting the locations and installation densities of stream gauge stations, which results in numerous problems, including regional imbalances and overlapping. To address these issues, a stream gauge network was constructed in this study considering both the transinformation of entropy (objective function 1) and the importance of each stream gauge station (objective function 2). To account for both factors, the optimal combinations that satisfied the two objective functions were determined using the Euclidean distance. Based on the rainfall runoff analysis results, unit hydrographs reflecting stream connectivity were derived and applied to entropy theory. The importance of each stream gauge station was calculated considering its purposes, such as flood control, water use, and environment. When this method was applied to the Namgang Dam Basin, it was found out that eight out of 12 stream gauge stations were required. The combination of the selected stations reflected both the transinformation of entropy and the importance of each station.

## 1. Introduction

Unpredictable weather conditions occurred worldwide in recent years. To improve adaptability to such weather conditions and to establish countermeasures, it is necessary to obtain hydrological data by designing reliable hydrometric networks. Among the various structures used to obtain hydrological data, stream gauge stations are important for measuring the water levels of streams and understanding their flow. Thus, stream gauge networks must be designed to achieve an understanding of basin water resources [1], and emphasis is placed on methods of obtaining and managing consistent hydrological data through the efficient construction of stream gauge networks based on manpower and budget limitations.

The concept of evaluating the design and construction of stream gauge networks was established since the 1980s. Stream gauge stations are usually evaluated using entropy theory, principal component regression analysis, and correlation analysis, although the existing stream gauge networks are mainly evaluated using entropy theory [2,3,4,5,6,7]. Al-Zahrani and Husain [2] applied the entropy concept to the optimal number of stream gauge stations in a dense network and to the expansion of a stream gauge network with a low density. Caselton and Husain [3] applied the concept of information transmission to the design of a hydrometric network, and Chapman [4] evaluated the reduction in uncertainty of hydrological data using entropy. Later, Krstanovic and Singh [5] evaluated the spatial variability of rainfall using entropy and examined the suitability of the stream gauge network in Louisiana. Yang and Burn [6] subsequently attempted to design an optimal stream gauge network using the entropy method. Moreover, Joo et al. [7] derived unit hydrographs using empirical formulas and actual runoff data and showed that it is appropriate to use actual runoff data that can reflect stream connectivity when applying entropy theory.

Regarding the other stream gauge station evaluation methods, Kyung et al. [8] optimized a stream water quality monitoring network using the Kriging technique and the branch and bound method. Moreover, Theodossiou et al. [9] applied the Kriging technique to the optimization of a groundwater monitoring network and quality improvement of the acquired data. The Ministry of Land, Infrastructure, and Transport of South Korea (MOLIT) [10] constructed a national stream gauge network by performing both a status survey and a field survey from 2006 to 2007. Alfonso et al. [11] proposed a method of placing stream gauge stations based on information theory measurement, and Putthividhya and Tanaka [12] evaluated the importance of each rain gauge for the Yom river basin in Thailand using multivariate geostatistical algorithms that integrated altitude, humidity, and temperature data. Nguyen et al. [13] proposed an optimal basin hydrology network using the relative distances between rain gauges, spatial interpolation of rainfall, and multi-layer analysis based on a geographic information system (GIS) to develop a real-time flood warning system for the Thu Bon river basin in Vietnam. Chacon-Hurtado et al. [14] mentioned the importance of sensor networks for water quality, stream gauge stations, and flood early warning systems and proposed statistics- and information-theory-based methods for evaluating sensor networks in terms of rainfall runoff and hydrological modeling. In addition, there are studies that improved and developed the monitoring of groundwater and surface water using MRMR (Minimum-Redundancy and Maximum-Relevance criteria) and Akaike information criterion theory [15,16,17]. Many studies were also conducted to optimize environmental monitoring including the atmosphere using GIS, root-mean-square error (RMSE), correlation analysis, and principal component analysis [18,19,20]. 

Entropy theory is applied to various water resource areas. Singh [21] described a process for parameter estimation based on entropy in hydrology, and Chou [22] proposed a new method of analyzing the complexity of the runoff coefficient for rainfall, runoff, and time using multi-scale entropy. Zhu et al. [23] described the evolution of water resource systems from the perspectives of climate change and human involvement using entropy theory. Wrzesiński [24,25] and Faiz et al. [26] assessed the uncertainty of flow regime characteristics and precipitation variability using entropy theory.

In addition, attention needs to be paid to the work of the World Meteorological Organization (WMO) [27] and Wahl [28], who performed studies on calculating the ratings of stream gauge stations. The WMO [27] proposed determining the control area of each stream gauge station by calculating the minimum observation density according to the geographical conditions, but this approach had limitations in terms of reflecting the upstream and downstream runoff characteristics for stream gauge stations. Wahl [28] quantitatively evaluated stream gauge stations using six evaluation items: the characteristics of each point, water use, problems related to water resources, data use from a water resource planning perspective, data use from a water resource management perspective, and economic efficiency. The work of Wahl [28] was extended by MOLIT [29] and was used to evaluate the importance of stream gauge stations in South Korea based on their installation purposes. 

Many problems frequently occur since the evaluation method does not include clear criteria for the placement of stream gauge stations. The current evaluation method of the stream gauge network is applied on a point-by-point basis. It causes a lot of problems, including the concentration of stations and overlapping observations in some regions due to installation purposes. Many entropy theories were applied in previous studies. This is because the entropy theory can construct a stream gauge network that takes into account the characteristics between the watersheds where the stations are located. Because stream gauge stations have a high linkage between watersheds (upstream and downstream), analysis using only water level data, such as correlation analysis or regression analysis, is inaccurate. In order to apply correlation analysis or regression analysis, variables (water level observation data) must be independent. Due to the independence of data, these methodologies such as correlation analysis cannot be used. Because correlation analysis or regression analysis does not reveal the impact of water level data between watersheds, it is not possible to construct an optimal stream gauge network. 

However, the evaluation of stream gauge networks using entropy theory assesses such networks only based on the amount of transinformation; it has a limit taking into consideration only characteristics of the observed data and watershed. In addition, previous studies were focused only on selecting the optimal combination of the existing stream gauge networks, and the importance of such networks according to the installation purposes of the stations could not be considered. For example, important stream gauge stations for flood control and water use may be excluded from optimal stream gauge networks because their transinformation of entropy is small. The optimal stream gauge network should account for both the acquisition of data that represent the basin and the installation purposes of the stream gauge stations. In other words, the entropy theory shows the hydrological similarity of water level data between water level stations. In addition, the stage gauge station rating shows the importance of the station.

In this study, stream gauge networks were constructed considering both the transinformation of entropy (objective function 1) and the importance of each stream gauge station (objective function 2). To calculate the entropy parameters that reflect stream connectivity, unit hydrographs were derived using actual rainfall runoff data and were converted into a probability density function. Regarding the importance of each stream gauge station, the rating of each station was calculated considering its purposes, such as flood control, water use, and environment, and this method was applied to the Namgang Dam Basin. To include both the maximum transinformation of entropy and the importance of each stream gauge station, the optimal combinations that satisfied both objective functions were determined using the Euclidean distance. The remainder of this report is organized as follows: in Section 2, the basic theories for the methodologies used in this study are introduced. Section 3 describes the application of these methodologies to the study area and discusses the results. Finally, Section 4 summarizes the conclusions of this study.

## 2. Basic Theories

### 2.1. Entropy Theory

Entropy is generally known as a measure of disorder or uncertainty, but it is defined as the information capacity of a signal in information theory [30]. When a signal is transmitted in the course of information exchange, the uncertainty of the signal is reduced if the information capacity of the signal is sufficiently large. Therefore, the information capacity of the signal can be indirectly measured based on the degree of uncertainty reduction [31]. Shannon and Weaver [30] defined the marginal entropy for the discrete random variable *X* as follows:

(1) $$H\left(X\right)=-\overset{N}{\underset{n=1}{\sum }}P\left(x_{n}\right)lnP\left(x_{n}\right),\;\;\;\;\;\;n=1,2,3,\ldots ,N,$$

where $P\left(x_{n}\right)$ is the probability of the occurrence of $x_{n}$, and the marginal entropy H(x) means the amount of information or the uncertainty of x. When $y_{m}$(m=1, 2, ⋯, N) related to the random variable $x_{n}$ exists, the uncertainty of $x_{n}$ can be reduced if $x_{n}$ is estimated using $y_{m}$. Based on this principle, the uncertainty of the random variable X left by the given variable Y can be estimated as follows:

(2) $$H\left(X|Y\right)=-\overset{N}{\underset{n=1}{\sum }}\overset{N}{\underset{M=1}{\sum }}P\left(x_{n},y_{m}\right)lnP\left(x_{n}|y_{m}\right),$$

where $P\left(x_{n,}y_{m}\right)$ is the joint probability of $X=\left(x_{n}\right)$ and $Y=\left(y_{m}\right)$, and $P(x_{n}|y_{m})$ is the conditional entropy of X for the given Y, which also represents the amount of information lost during the information transfer between X and Y [6]. Based on the given Y, the degree of reduction in the uncertainty of X or the amount of information transferred between X and Y is as follows:

(3) T(X,Y)=H(X)−H(X|Y).

Thus far, the probability density functions that can be applied to the entropy method include the normal, log-normal, and Gamma distributions, and the application of other distribution types is limited. This limitation exists because entropy values are theoretically derived only for the three abovementioned distribution types, and complex multidimensional numerical integration is required for the other types [6]. The concept of entropy is applicable to hydrological time series data. When it is assumed that the continuous random variable X follows the probability density function f(x), the range of X can be divided by the interval Δx. Chapman [4] defined the marginal and conditional entropy using the interval Δx/x, which is proportional to the range of the variable, instead of the fixed interval Δx, as follows:

(4) $$H\left(X;\Delta x\right)=u+0.5ln\left(2\pi e\sigma_{z}^{2}\right)-ln\left(\Delta x\right),$$

(5) $$H\left(X|Y;\Delta x\right)=u+0.5ln\left[\left(2\pi e\sigma_{z}^{2}\right)\left(1-\rho_{zw}^{2}\right)\right]-ln\left(\Delta x\right),$$

where $\mu_{z}$ and $\sigma_{z}$ are the mean and standard deviation of z(=lnx), and $\rho_{zw}$ is the correlation coefficient between z and w (=lny). 

The optimization of a stream gauge network present in a basin means that the number of stream gauge stations is reduced so that the overlapping information between the stations can be minimized and the information about the basin obtained from the maintained stations is maximized. In other words, the maximum information about the basin must be obtained from the minimum number of stations. Thus, the objective function of optimization can be expressed as follows [2]:

(6) $$MAX\;\left[T\left(X_{1},X_{2},\ldots X_{m};X_{k},X_{l},\ldots X_{p}\right)\right],$$

where m is total number of stream gauge stations currently in the basin, and p is the number of stations to be maintained. Therefore, $T\left(X_{1},X_{2},\ldots \ldots .X_{m};X_{k},X_{l},\ldots ..X_{p}\right)$ means the information about the basin that can be obtained from p stations, which can be expressed as shown in Equation (7).

(7) $$MAX\;\overset{m}{\underset{i=1}{\sum }}T\left(X_{i};X_{k},X_{l},\ldots X_{p}\right)=MAX\left(H\left(X_{k}\right)+H\left(X_{p}\right)+\overset{m-p}{\underset{i=1}{\sum }}\overset{p}{\underset{j=k}{\sum }}T\left(X_{i},X_{j}\right),\;i\neq j\right),$$

where $H\left(X_{k}\right)+\cdots +H\left(X_{p}\right)$ is the sum of the marginal entropy of each selected station, and $\overset{m-p}{\underset{i=1}{\sum }}\overset{p}{\underset{j=k}{\sum }}T\left(X_{i},X_{j}\right)$ is the amount of information transferred between the selected and unselected stations or the amount of information about the unselected stations that can be obtained from the selected stations. As the number of selected stations increases, the amount of information that can be obtained will increase. After a certain time point, however, the amount of information that can be obtained from the selected stations decreases due to the amount of overlapping information between stations. Therefore, the optimal stream gauge network is the combination of stream gauge stations that can maximize the amount of information about the basin [7].

### 2.2. Stream Gauge Network Grading Methodology

Wahl [28] evaluated stream gauge stations by determining the importance of each station using six items (item 1: characteristics of the point, item 2: water use for various purposes, item 3: problems related to water resources, item 4: data use from a water resource planning perspective, item 5: data use from a water resource management perspective, and item 6: economic efficiency). For item 1, six characteristics of each point were examined, including flow rate, basin, and data. For item 2, the water use was designated as domestic, industrial, or agricultural. Item 3 was related to water quality and, thus, was evaluated using the presence of water quality monitoring points in the vicinity, data length, and unmeasured rate. For items 4 and 5, the regional importance and importance from a management perspective were evaluated. For item 6, each stream gauge station was evaluated based on the utilization of domestic, industrial, and agricultural water. 

The work of Wahl [28] was extended by MOLIT [29]. MOLIT [29] categorized installation purposes by modifying the evaluation items of Wahl [28] according to the current hydrological situation and proposed management measures for each installation purpose. To calculate the rating of each stream gauge station, the flood control, water use, and environmental purposes of each station, as well as whether it was a national hydrologic observation station, were considered. Here, flood control refers to the forecasting, control, and prevention of flooding, and water use refers to water resource management/supply and water-related conflict factors. The environmental factor describes the operation of stations as points with total maximum daily loads in effect. In this study, the importance of each stream gauge station was evaluated by referring to the installation purposes of stream gauge stations proposed by MOLIT [29] to determine the rating of a stream gauge network. 

### 2.3. Euclidean Distance

The most important thing in the optimal combination of two objectives is the weight between the objective functions [32]. If the weights between the objective functions are the same, the distance measurement technique can be the most intuitive and efficient way of multi-objective optimization. Several optimization methods (e.g., Pareto optimization techniques, weighting methods, etc.) cannot be said to be innovative within the same weight. Distance measurement techniques have various methodologies such as Euclidean, City Block, Chebyshev, Minkowski, Quadratic, and Canberra. In this paper, the Euclidean distance method, which is most commonly used, was applied. 

The optimal combinations that satisfied the two objective functions were determined using the Euclidean distance to consider both the maximum transinformation of entropy and the installation purposes of each stream gauge station. Various optimization techniques, such as genetic algorithms and harmony searches, can be utilized to find the optimal combinations, but the enumeration technique was used in this study to consider all cases. The enumeration technique, which is a local optimization technique, is the most primitive means of finding the optimal solution. The enumeration technique was chosen because the optimal combinations were determined based on rankings and, thus, the combinations of all stations were required.

The optimization technique is mainly applied to cases with single objective functions. Although multiple functions can be optimized independently, it is not easy to obtain a solution that simultaneously achieves multiple purposes. This topic is consistently an issue in operations research. The optimal solution for all objective functions basically does not exist in many cases. Therefore, in this study, a distance measurement technique was used to determine the optimal combinations that satisfied the two objective functions. 

In general, distance measurement techniques are used to represent the degree of similarity between objects quantitatively for cluster analysis, which is a data mining technique. The Euclidean distance is the geometric distance in a multidimensional space and can be calculated as follows:

(8) $$d_{e}=\;\sqrt{\overset{n}{\underset{i=1}{\sum }}{\left(x_{i}-y_{i}\right)}^{2}}.$$

**Figure 1:**
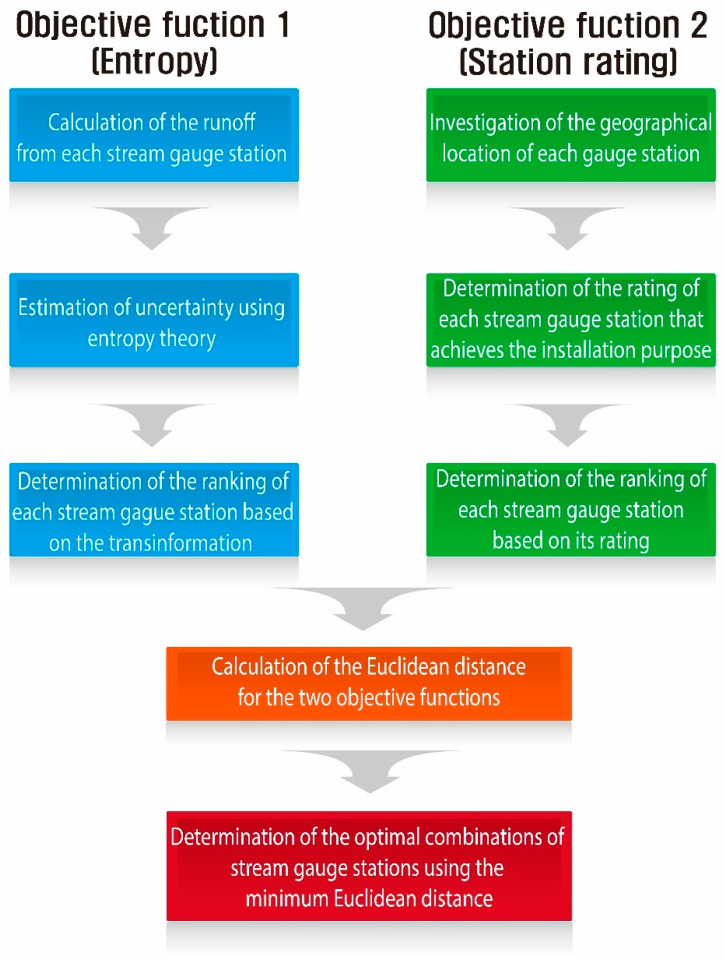
Procedure for integrating two objective functions.

Figure 1 shows the application process of calculating the Euclidean distance.

## 3. Application

### 3.1. Target Basin and Data Collection

**Figure 2:**
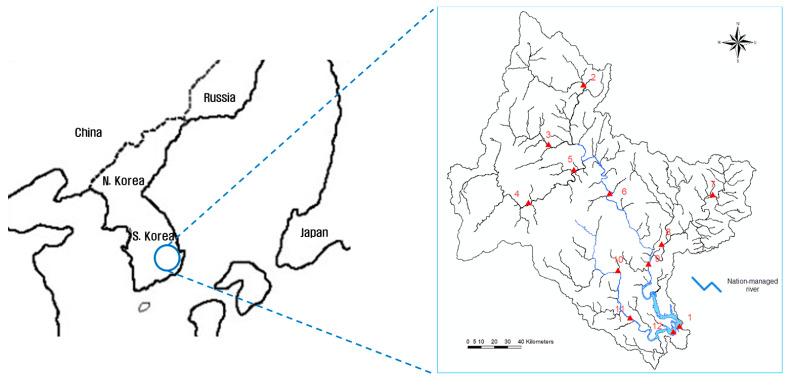
Study area and locations of stream gauge stations.

**Figure 3:**
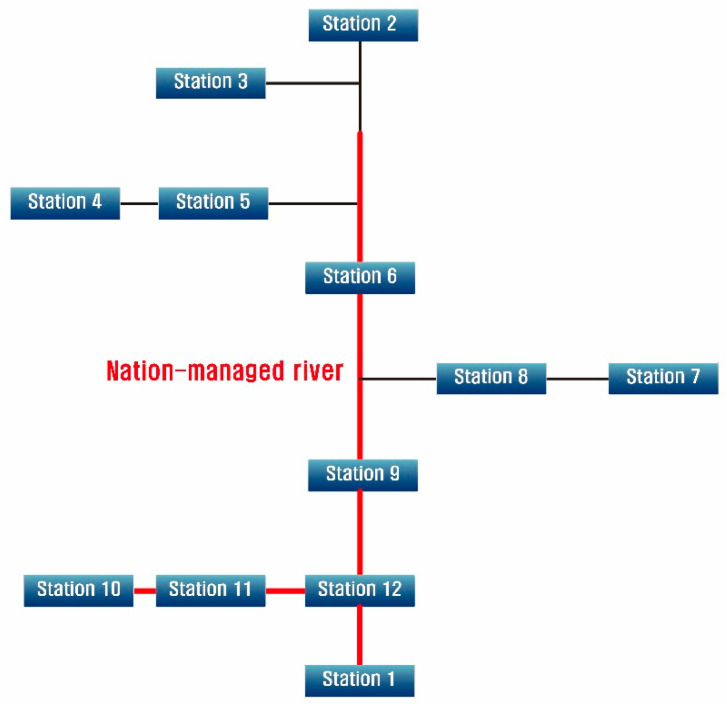
Schematic diagram of stream gauge stations connected by streams.

The Namgang Dam Basin, which was the target area of this study, is located in the southeast of the Korean peninsula and ranges from 35°00′ to 35°46′ north (N) in latitude and from 127°29′ to 128°28′ east (E) in longitude. The basin area and stream length are 2285 km^2^ and 110 km, respectively. Moreover, the Namgang Dam Basin has the largest area among the basins of Nakdong River, which is a representative national stream of South Korea. The installation locations of stream gauge stations are important for the basin because it is located in the upstream area of Namgang River, which flows into the main stream of Nakdong River. In the Namgang Dam Basin, 12 stream gauge stations are in operation. Figure 2 and Figure 3 show the locations of the stream gauge stations in the target basin and a schematic diagram of the stream connections, respectively. Table 1 summarizes the geographical characteristics of the study area.

### 3.2. Estimation of Uncertainty Using Entropy Theory

#### 3.2.1. Rainfall Runoff Analysis for Unit Hydrograph Derivation

To apply entropy theory, a unit hydrograph must be derived for each stream gauge station. In South Korea, Clark’s watershed routing method is mainly used. For the concentration, time, and storage constant parameters, empirical formulas are used, or the values are calculated by analyzing observational data. Joo et al. [7] compared unit hydrographs when empirical formulas and measurement data were employed and mentioned that the unit hydrographs derived using measurement data have the advantage of reflecting upstream and downstream runoff. Therefore, in this study, unit hydrographs were derived using measurement data to reflect the upstream and downstream runoff characteristics of each stream gauge station. To derive unit hydrographs using measurement data, it was firstly necessary to divide each stream gauge station into sub-basins and to extract geomorphological factors to identify the runoff characteristics of each sub-basin. In this study, the sub-basins were constructed, and geomorphological factors were extracted by linking the HEC-GeoHMS and HEC-HMS models. For the rainfall runoff analysis using the HEC-HMS model, the NRCS (Natural Resources Conservation Service) method was applied as a rainfall loss model, Clark’s unit hydrograph method was utilized as a watershed routing method, and the Muskingum method was employed as a channel routing method. In addition, rainfall data were used for hourly data provided by the Korea Meteorological Administration.

**Figure 4:**
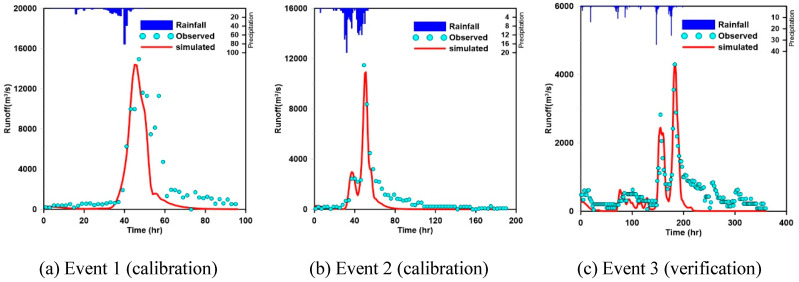
Rainfall runoff analysis results for parameter calibration and verification.

The continuous Kraven (II) formula and Sabol formula were used to obtain the initial values for calculating the parameters of the Clark unit hydrograph, and the Thiessen method (application of 16 rainfall observatories) was applied to calculate the average rainfall in the area. The runoff parameters were determined using the rainfall runoff events called event 1 (Rusa) and event 2 (Meami), and the appropriateness of the calculated runoff parameters was verified through the rainfall runoff event called event 3. Table 2 and Figure 4 show the characteristics of the rainfall events employed for the calibration and verification of the runoff parameters and the results of performing calibration and verification for the target basin.

#### 3.2.2. Derivation of Unit Hydrographs by Station and Their Conversion into a Probability Density Function

**Figure 5:**
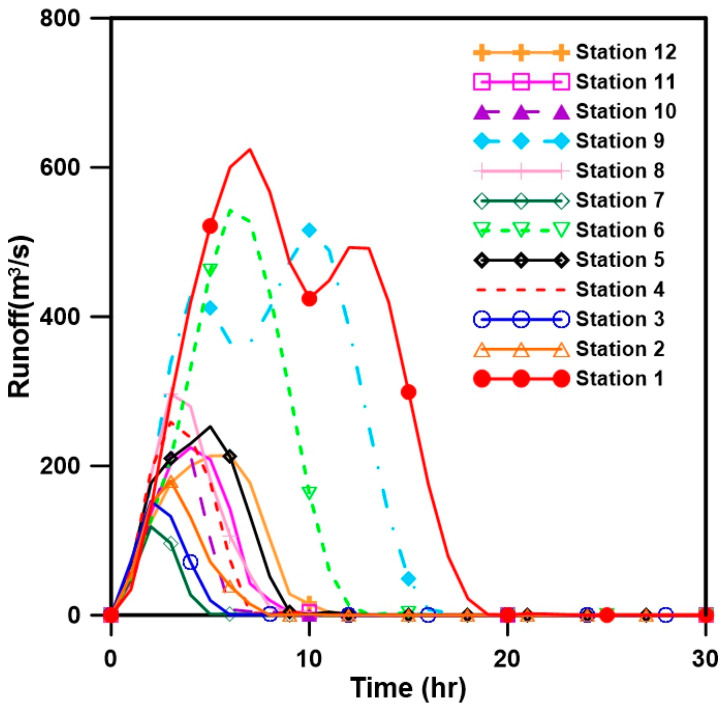
Derived unit hydrographs for the individual stream gauge stations.

To construct a stream gauge network by applying entropy theory, unit hydrographs must be derived for each point, and a probability density function capable of adequately expressing the derived unit hydrographs must be determined. In this study, unit hydrographs were derived by analyzing rainfall runoff events, and the probability density function parameters were determined through the optimization process. Figure 5 shows the derived unit hydrographs. As can be seen from the figure, the unit hydrographs calculated based on the actual rainfall runoff events do not show smooth hydrograph patterns. This is due to the fact that the upstream and downstream runoff characteristics were reflected, and the runoff of the downstream area was directly affected by that of the upstream area.

To construct a stream gauge network using entropy theory, a probability density function capable of adequately expressing the derived unit hydrographs by station must be determined. The probability distribution types used to analyze hydrological data are largely divided into discrete distributions and continuous distributions. Among the discrete distributions, binominal and Poisson distributions are frequently used to determine the time intervals of rainfall or flooding with a certain magnitude or the occurrence probabilities of certain events. Most hydrological phenomena, however, occur continuously and, thus, continuous distribution types are mainly used for the probabilistic analysis of such phenomena. Among the continuous distributions, normal, log-normal, Gamma, log-Pearson, and generalized extreme value distributions are the most frequently used for hydrological analysis [7,33,34]. Entropy equations that are known to follow probability density functions include the normal, log-normal, and Gamma distributions. In this study, the log-normal distribution that was the most suitable for the unit hydrographs derived for each sub-basin was applied, and it can be expressed as follows [7]:

(9) $$f\left(x\right)=\frac{1}{xb\sqrt{2\pi }}\frac{1}{x}\exp\left[-\frac{1}{2}{\left(\frac{lnx-\mu_{y}}{\sigma_{y}}\right)}^{2}\right],\;0\leq x<\infty ,$$

where lnx=y, μ = mean, and σ = standard deviation. To estimate the parameters of a log-normal distribution, the moment method is generally used. In this study, however, the parameters were determined through the optimization process (the Visual-Basic program was used) for greater accuracy. Table 3 summarizes the probability density function parameters calculated using the optimization process. Average and SD (standard deviation) were parameters of the probability density function used to derive Clark unit hydrograph and log-normal distribution. Table 4 shows the results of calculating the transinformation of entropy theory using the calculated probability density function. In Table 4, “sum” means the total transinformation of each stream gauge station.

For the information matrix calculated in Table 5, the maximum transinformation for each combination of stations was calculated. The results are shown in Table 6. In this case, the optimal stream gauge networks were constructed considering only the change in the transinformation of data, and they were the optimal stream gauge networks for the purpose of providing hydrological data. In other words, they are not the networks to be employed for purposes such as flood control, water use, and environmental objectives. Moreover, these results were used as the first objective function of the optimal stream gauge network, and the ultimate purpose of the first objective function was to obtain hydrological data that represent the basin.

### 3.3. Determination of the Rating of Each Stream Gauge Station

The ratings of the stream gauge stations were determined by referring to their installation purposes according to MOLIT [28]. Table 1 shows the evaluation criteria for each item according to the installation purposes proposed by MOLIT (Table 6).

For the installation purposes mentioned by MOLIT [28], i.e., flood control, water use, environmental purposes, and presence of nation-managed streams, the stations that served all four purposes were assigned the first rating, and those that served three purposes were given the second rating. Similarly, the stations that served two purposes were given the third rating, and those that served only one purpose were assigned the fourth rating. Table 7 shows the ratings of the stream gauge stations in the target basin classified according to these criteria. To quantify the calculated ratings, 10 points were given to the first rating, 7.5 points to the second rating, 5.0 points to the third rating, and 2.5 points to the fourth rating. The importance of each combination of stations according to their purposes was evaluated by adding the rating points of the selected stations. In other words, if the selected stations had the first and second ratings, the importance value was 17.5 (10 + 7.5). These results were used as the second objective function to reflect the importance of each station according to its purpose.

In this study, each installation purpose was treated equally when calculating the ratings of the stream gauge stations. For example, although a stream gauge station is installed for both flood control and water use purposes, flood control can be more important for some stations. Therefore, when the same weight is assigned to each purpose, issues can arise with accurately identifying the installation purposes of the stream gauge stations. In other words, in the case of the Namgang Dam Basin, although all four purposes, i.e., flood control, water use, environmental purposes, and presence of nation-managed streams, are served at the same time, the weight of each purpose must be classified according to the main purpose of each station. However, evaluation of the installation purposes of each station exceeded the scope of this study.

### 3.4. Construction of Optimal Stream Gauge Networks Using Euclidean Distance

All of the combinations were presented using the enumeration technique to consider both the entropy results that included the transinformation of the observation data and the importance of each stream gauge station according to its installation purpose. In total, there were 4095 possible combinations of the 12 stations in the study area (Table 8).

The Euclidean distance was employed to select the optimal stream gauge network combinations. For this purpose, the total transinformation and the importance according to installation purpose were calculated for all combinations of stations, and re-scaling was performed to normalize the calculated values to the range of 0 to 1, as shown in Equation (10).

(10) $$\mathrm{Standardization}\left(0-1\right)=\frac{\left(x_{i}-x_{\min}\right)}{\left(x_{\max}-x_{\min}\right)},$$

where $x_{i}$ is the characteristic data value, $x_{\max}$ is the maximum value among all of the data, and $x_{\min}$ is the minimum value among all of the data. Therefore, the importance according to the transinformation and installation purposes of each combination ranged from 0 to 1. When the ranking of the total transinformation (*x*-axis) and that of the importance according to installation purpose (*y*-axis) were expressed for each combination, each combination had its own coordinate values, and 4095 points were obtained in total. Moreover, the total transinformation rankings did not overlap because the amount of entropy information selected according to the number of stations did not overlap. On the other hand, the importance rankings according to installation purpose did overlap depending on the combinations of stations because the importance was classified using four ratings (10 points for the first rating, 7.5 points for the second rating, 5.0 points for the third rating, and 2.5 points for the fourth rating, as mentioned previously). When the two objective functions were integrated using the Euclidean distance, however, the optimal network evaluation could be performed without any significant problems because the entropy rankings were different even though the importance rankings according to installation purpose were identical. These issues will be considerably reduced if the installation purpose ratings are diversified in the future. 

**Figure 6:**
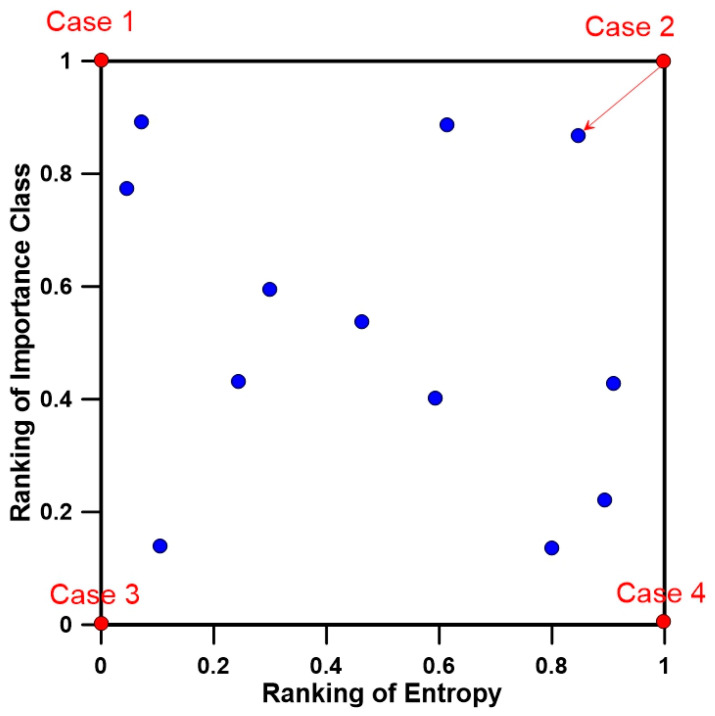
Integration of two objective functions using the Euclidean distance.

Figure 6 shows the process of selecting the combinations of stations using the Euclidean distance. For Case 1, the importance according to installation purpose is high, but the representativeness for the basin is low. For Case 4, the representativeness of the basin is excellent due to large amount of transinformation, but the importance according to installation purpose is not sufficient. Case 3 is the worst case because it does not satisfy the transinformation of stream gauge station and the importance of each installation purpose. Case 2 is the best case because both the amount of transinformation and the importance according to installation purpose are satisfied simultaneously. However, it is practically difficult to satisfy both criteria perfectly. Therefore, it is efficient to select the combination that is the closest to Case 2 as the optimal stream gauge network, which can be determined using the Euclidean distance. 

## 4. Results and Discussion

**Figure 7:**
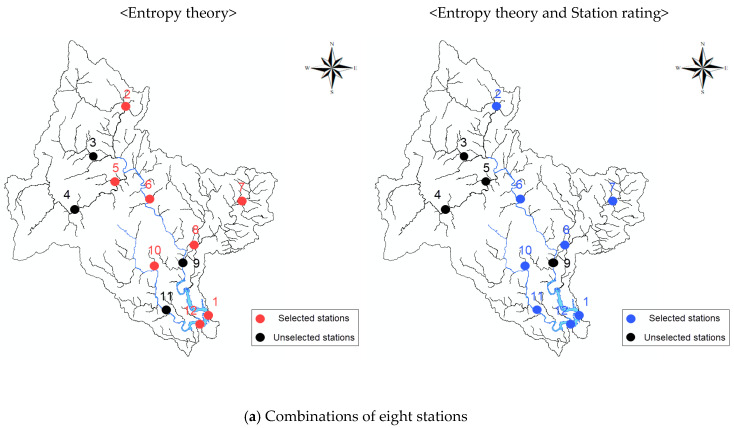

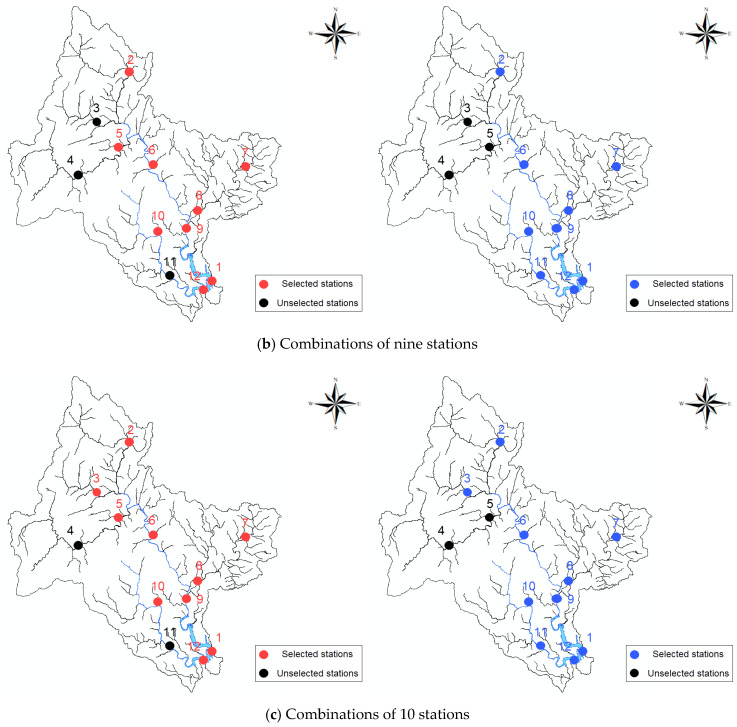
Comparison of the optimal stream gauge networks determined using each method.

Table 9 presents the results of applying the methodology of Figure 6 to the target basin, showing the minimum Euclidean distances for all combinations of stations according to the number of stations. Figure 7 depicts the results of selecting 8–10 stations using two methodologies. When only the proposed methodology and entropy theory were applied, the maximum transinformation was obtained when 10 stations were selected, but the combinations of stations differed depending on the methodology. Moreover, there was no significant difference in the amount of transinformation between the eight stations selected. In particular, when two objective functions were applied, the transinformation differed by 2.21, further reducing the difference. Therefore, it can be said that a sufficient amount of transinformation and importance according to installation purpose can be achieved through the operation of eight stream gauge stations. From an economical perspective, it is efficient to determine eight stream gauge stations (1, 2, 6, 7, 8, 10, 11, 12) by applying the two objective functions (Table 9). 

For the one-station combination, station 4 was selected when only entropy theory was applied, but station 8 was selected when two objective functions were applied. Station 4 was not selected when both objective functions were applied because it was given the third rating even though it showed the maximum transinformation. Station 8, on the other hand, was selected as the optimal stream gauge station when both objective functions were applied because it was given the second rating even though it had the second ranking in terms of transinformation. Regarding the combinations of 8–10 stream gauge stations in the category of the optimal stream gauge network, station 5 was selected when only entropy theory was applied, but station 5 was removed and station 11 was selected for all corresponding combinations when both objective functions were applied. It was found that a station with a lower rating was replaced with a station with a higher rating, considering that station 5 with the third rating was replaced with station 11 with the second rating, indicating that the use of two objective functions properly reflected the ratings of stations in constructing the optimal stream gauge networks. The use of only entropy theory did not yield a large difference with the use of both objective functions in terms of constructing the optimal stream gauge networks. If stream gauge networks are evaluated using the proposed methodology for different basins, however, the results will be different.

However, some limitations of this study should be noted. For instance, the same weight was assumed for both objective functions (the amount of hydrological information calculated using entropy theory and the importance according to installation purpose). There are cases in which the stream gauge station purposes of flood control and water use are more important than producing stable hydrological data, depending on the basin. In such cases, it is necessary to assume different weights for different objective functions, as well as to quantitatively identify the factors that determine the importance of installation purposes through various methodologies. As the purpose of this research was to develop a method of evaluating stream gauge networks that satisfy various purposes by integrating objective functions, the evaluation of each stream gauge station was outside the scope of this study. If each stream gauge station is evaluated in the future by investigating the characteristics for various basins, the weights of the objective functions will be more objective. It is also necessary to perform further studies of various factors for determining the importance of stream gauge stations.

## 5. Conclusions

In this study, stream gauge networks were evaluated with the focus of constructing a stream gauge network capable of providing the maximum transinformation of entropy and the importance of the stream gauge stations according to installation purpose. To this end, unit hydrographs were derived pointwise by analyzing runoff observation data, and they were applied to entropy theory through conversion into a probability density function. Moreover, the ratings of stream gauge stations presented by MOLIT [20] were used to designate the importance according to installation purpose. The combinations of the transinformation of entropy and the importance of the stream gauge stations were found through the enumeration technique, and the optimal combinations were determined using the Euclidean distance. The results of applying this procedure to the Namgang Dam Basis are summarized below. 

When stream gauge networks were evaluated using the proposed methodology, different networks were constructed compared to when only entropy theory was applied. This is a better result that reflects the importance of the stations according to their installation purposes. It was found that eight out of 12 stream gauge stations are required for the Namgang Dam Basin, which was the target basin. This result reflects both the importance of each stream gauge station according to its installation purpose and the characteristics of the data. In the future, it will be necessary to consider various factors related to the installation purposes to determine the importance of each stream gauge station. Moreover, to evaluate the importance of each station, detailed examinations of the various installation purposes of the stations, their accessibility, and the flood hazard zones, as well as further research regarding their weights, are required. The methodology employed in this study confirmed that entropy theory is not just an information theory; rather, it can be utilized for basin management. It is expected that the results of this study will facilitate the construction of optimal stream gauge networks that can serve various installation purposes.

## Figures and Tables

**Table 1:** Geomorphological characteristics of the study area. N—north; E—east.

# of Stream Gauge Station	Name of Stream Gauge Station	Basin Area (km^2^)	Stream Length (km)	Stream Slope	Shape Factor	Latitude (N)	Longitude (E)
1	Namgang Dam	2293.4	111.2	0.00411	0.185	35°09′42″	128°02′08″
2	Aneui	161.8	31.7	0.01523	0.214	35°37′39″	127°48′55″
3	Hamyang	122.0	22.2	0.02311	0.251	35°30′49″	127°43′54″
4	Macheon	218.0	31.3	0.00937	0.322	35°24′06″	127°40′58″
5	Imcheon	432.0	47.2	0.00936	0.206	35°27′52″	127°47′28″
6	Sancheong	1134.0	67.2	0.00672	0.249	35°25′04″	127°52′27″
7	Samga	104.0	13.3	0.00681	0.571	35°24′49″	128°07′03″
8	Shinan	413.0	23.9	0.08452	0.723	35°19′12″	127°59′45″
9	Mukgokgyo	1709.2	89.3	0.05462	0.214	35°16′55″	127°57′51″
10	Taesu	143.0	28.3	0.03724	0.304	35°16′12″	127°53′33″
11	Changchon	336.0	40.0	0.01882	0.205	35°10′43″	127°55′13″
12	Naepyeong	2293.0	45.7	0.00710	1.098	35°09′04″	128°01′16″

**Table 2:** Rainfall events for rainfall runoff calibration and verification. Max.—maximum; Ave.—average.

# Event	Date	Total Rainfall (mm)	Rainfall Duration (h)	Max. Rainfall Intensity (mm/h)	Ave. Rainfall Intensity (mm/h)	Note
1	30 August 2002	248	50	81	4.7	Calibration (Rusa)
2	11 September 2003	166	53	20	3.1	Calibration (Meami)
3	23 July 2009	203	198	34	1.0	Verification

**Table 3:** Parameters estimated for the log-normal distribution.

Stream Gauge Station Number	Average	SD
1	2.280	0.492
2	1.445	0.351
3	1.255	0.355
4	1.507	0.340
5	1.665	0.436
6	2.023	0.299
7	1.208	0.311
8	1.560	0.342
9	2.131	0.522
10	1.501	0.299
11	1.619	0.365
12	1.828	0.405

**Table 4:** Information matrix for all stream gauge stations.

	1	2	3	4	5	6	7	8	9	10	11	12	Sum
**1**	**5.32**	0.08	0.03	0.11	0.26	0.80	0.01	0.14	1.40	0.09	0.19	0.48	**8.91**
**2**	0.08	**4.98**	1.00	2.01	1.03	0.09	0.79	1.44	0.20	1.68	1.12	0.48	**14.9**
**3**	0.03	1.00	**4.99**	0.72	0.48	0.02	2.22	0.56	0.09	0.63	0.46	0.20	**11.4**
**4**	0.11	2.01	0.72	**4.95**	1.29	0.13	0.56	2.21	0.25	2.42	1.55	0.62	**16.82**
**5**	0.26	1.03	0.48	1.29	**5.19**	0.34	0.37	1.58	0.51	1.11	2.08	1.20	**15.44**
**6**	0.80	0.09	0.02	0.13	0.34	**4.82**	0.01	0.18	0.99	0.11	0.27	0.71	**8.47**
**7**	0.01	0.79	2.22	0.56	0.37	0.01	**4.86**	0.43	0.06	0.50	0.35	0.14	**10.30**
**8**	0.14	1.44	0.56	2.21	1.58	0.18	0.43	**4.95**	0.32	1.99	2.19	0.78	**16.77**
**9**	1.40	0.20	0.09	0.25	0.51	0.99	0.06	0.32	**5.37**	0.23	0.41	0.89	**10.72**
**10**	0.09	1.68	0.63	2.42	1.11	0.11	0.50	1.99	0.23	**4.82**	1.41	0.57	**15.56**
**11**	0.19	1.12	0.46	1.55	2.08	0.27	0.35	2.19	0.41	1.41	**5.02**	1.03	**16.08**
**12**	0.48	0.48	0.20	0.62	1.20	0.71	0.14	0.78	0.89	0.57	1.03	**5.12**	**12.22**
**Sum**	**8.91**	**14.9**	**11.4**	**16.82**	**15.44**	**8.47**	**10.30**	**16.77**	**10.72**	**15.56**	**16.08**	**12.22**	**-**

**Table 5:** Results of the optimized stream gauge network (entropy theory).

No. of Stations	Optimized Combination of Stream Gauge Stations	Max. Information Content	Transinformation According to the Total Number of Stations
1	4	16.81	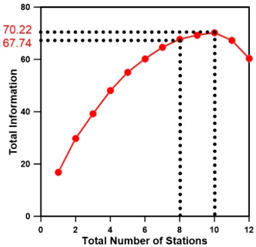
2	4, 11	29.77	
3	4, 9, 11	39.16	
4	3, 4, 9, 11	48.02	
5	3, 4, 5, 8, 9	55.09	
6	3, 4, 5, 6, 8, 9	60.24	
7	1, 2, 4, 6, 7, 11, 12	64.58	
8	1, 2, 5, 6, 7, 8, 10, 12	67.74	
9	1, 2, 5, 6, 7, 8, 9, 10, 12	69.27	
10	1, 2, 3, 5, 6, 7, 8, 9, 10, 12	70.22	
11	1, 2, 3, 5, 6, 7, 8, 9, 10, 11, 12	67.31	
12	1, 2, 3, 4, 5, 6, 7, 8, 9, 10, 11, 12	60.39	

**Table 6:** Importance evaluation criteria for stream gauge stations [20].

Purpose	Goal	Observation Points
Flood control	Flood forecasting	• Flood forecasting points • Flood forecasting model analysis points • Support or backup for the above major stream gauge stations related to flood forecasting
	Flood control facility	• Water level monitoring points of dams, reservoirs, or weirs with flood control functions
	Disaster prevention (disaster management)	• Points affecting the discharge of national dams • Important points for disaster management, such as bridge flood areas and flood-prone areas
Water use	Water resource management and supply	• Points with officially announced instream flows • Middle zone exit points
	Water right conflict	• Metropolitan city and province boundary points
Environment	Water quality, etc.	• Points with total maximum daily loads in effect
Contribution	National stream	• Stream gauge stations installed in national streams

**Table 7:** Ratings of stream gauge stations.

Number of Station	Name of Stream Gauge Station	Water Use	Flood Control	Environmental Purposes	Presence of Nation-managed Streams	Importance Rating
1	Namgang Dam	O	O	O	O	1
2	Aneui	O	O			3
3	Hamyang		O			4
4	Macheon		O	O		3
5	Imcheon		O	O		3
6	Sancheong	O	O	O	O	1
7	Samga		O			4
8	Shinan	O	O	O		2
9	Mokgokgyo		O	O	O	2
10	Taesu	O	O		O	2
11	Changchon	O	O		O	2
12	Naepyeong	O	O		O	2

**Table 8:** Numbers of combinations for 12 stream gauge stations.

Combination	Number of Possible Combinations
12C1	12
12C2	66
12C3	220
12C4	495
12C5	792
12C6	924
12C7	792
12C8	495
12C9	220
12C10	66
12C11	12
12C12	1
**Total**	**4095**

**Table 9:** Stream gauge network optimization results (entropy theory and station rating application). Min.—minimum.

No. of Stations	Optimized Combination of Stream Gauge Stations	Max. Information Content	Min. Euclidean Distance	Transinformation According to the Total Number of Stations
1	8	16.77	0.333	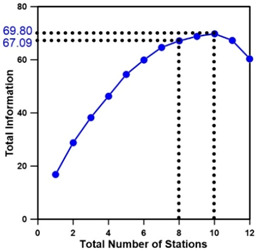
2	10, 11	28.82	0.177	
3	9, 10, 11	38.26	0.15	
4	1, 5, 8, 10	46.34	0.145	
5	1, 2, 10, 11, 12	54.44	0.131	
6	1, 3, 5, 6, 8, 10	59.91	0.132	
7	1, 2, 3, 6, 10, 11, 12	64.64	0.078	
8	1, 2, 6, 7, 8, 10, 11, 12	67.09	0.047	
9	1, 2, 6, 7, 8, 9, 10, 11, 12	68.81	0.046	
10	1, 2, 3, 6, 7, 8, 9, 10, 11, 12	69.80	0.107	
11	1, 2, 3, 5, 6, 7, 8, 9, 10, 11, 12	67.31	0.165	
12	1, 2, 3, 4, 5, 6, 7, 8, 9, 10, 11, 12	60.39	0.000	
